# 器官特异性转移肺癌细胞株的筛选及建立

**DOI:** 10.3779/j.issn.1009-3419.2014.03.20

**Published:** 2014-03-20

**Authors:** 清华 周, 玲玲 祖, 潞 李, 晓禾 陈, 晓峰 陈, 洋 李, 红雨 刘, 芝琳 孙

**Affiliations:** 1 610041 成都，四川大学华西医院，四川省肺癌分子重点实验室 Key Laboratory of Lung Cancer Molecular Biology, West China Hospital, Sichuan University, Chengdu 610041, China; 2 300052 天津，天津市肺癌转移与肿瘤微环境重点实验室，天津市肺癌研究所，天津医科大学总医院 Tianjin Key Laboratory of Lung Cancer Metastasis and Tumor Microenvironment, Tianjin Lung Cancer Institute, Tianjin Medical University General Hospital, Tianjin 300052, China

**Keywords:** 肺肿瘤, 器官特异性转移, 细胞株, 筛选, Lung neoplasms, Organ-specific metastasis, Cell lines, Screening and identification

## Abstract

**背景与目的:**

肺癌转移是肺癌的恶性标志和特征，也是肺癌病人治疗失败和死亡的主要原因。肺癌转移具有器官特异性，最常转移的部位是淋巴结、大脑、骨、肝脏和肾上腺。本研究的目的是应用我们实验室的人高转移大细胞肺癌细胞株L9981，筛选鉴定出具有器官特异性转移的肺癌亚代细胞株，为进一步研究肺癌细胞器官特异转移提供科学可靠的细胞模型。

**方法:**

通过裸鼠实验，将母系细胞株L9981-Luc皮下接种，每周一次动物活体成像观察肺癌器官转移情况，数周后构建出具有肺、脊柱、纵隔淋巴结和大脑等器官特异性转移的小鼠模型；处死裸鼠，切取肺、脊柱、纵隔淋巴结和大脑器官肺癌转移瘤进行原代培养，构建具有器官靶向特异性转移潜能的肺癌亚代细胞株；将第一代器官特异性转移肺癌细胞株接种裸鼠皮下，再次构建肺癌器官特异性转移小鼠模型；通过反复多次将肺癌器官特异性转移瘤构建肺癌细胞株，再裸鼠接种，最终获得具有肺、脊柱、纵隔淋巴结和大脑器官特异性转移的肺癌细胞株。

**结果:**

经过裸鼠反复接种，动物活体成像、动物体内反复筛选鉴定，成功构建了4株分别特异性转移到肺、脊柱、纵隔淋巴结和大脑的器官特异性转移肺癌细胞株，分别命名为L9981-LuM、L9981- BoM、L9981-LnM和L9981-BrM。

**结论:**

成功构建出具有肺、纵隔淋巴结、脊柱和大脑特异性转移的人大细胞肺癌细 胞模型，为进一步研究肺癌器官特异转移的分子机制、信号调控途径，以及未来研究和开发抑制或/和阻断肺癌转移的分子靶向药物提供了可靠的细胞模型。

## Abstract (en)

**Background and objective:**

Cancer metastasis is not only the malignant marker and characteristics, but also the main cause of failure to cure and lose their life in the patients with lung cancer. Lung cancer metastasis has organ-specific characteristics. The most common sites of lung cancer metastasis are mediastinal lymph node, brain, bone, liver and adrenal gland. The aim of this study is to screen and establish lung cancer cell model with organ-specific metastasis potential with human high-metastatic large cell lung cancer cell line L9981 established by our laboratory previously, and to provide cell models for studying the mechanisms and signal regulation of organ-specific metastasis of lung cancer.

**Materials and methods:**

The parent lung cancer cell line, L9981-Luc, was inoculated in the armpit of nude mice. The live animal imaging system, IVIS-200, was used to detect the lung cancer organ-specific metastasis every week. When the organ-specific metastasis were established, the nude mices bearing the lung cancer were sacrificed when they became moribund. Under sterile conditions, the organs (mediastinal lymph nodes, lung, spinal column and brain) with lung cancer organ-specific metastasis were removed and the metastasized nodules were dissected free of connective tissue and blood clots, and rinsed twice with medium. The metastasized nodules were finely minced using sterile scalpel blades in medium, and the cells were seeded in tissue culture dishes. Then, the cells with organ-specific metastasis potential were reinoculated into the armpit of nude mice, respectively. This processes were repeated to establish the organ-specific metastatic sublines of L9981-Luc cell line more than 10 times. Finally, the organ-specific metastasis sublines of L9981-Luc were screened and established, which the four cell lines have the characteristics only metastasized to brian, lung, bone and mediastinal lymph node.

**Results:**

A group of organ-specific metastasis cell lines which only metastasized to brian, lung, bone and mediastinal lymph node were successfully established through repeating reinoculatation, live animal imaging in nude mice, and screening and identification *in vitro*. We named the four cell lines as L9981-BoM, L9981-LuM, L9981-BrM and L9981-LnM, respectively. The L9981-BoM cell was only metastasized to bone. The l9981-LuM cell was only metastasized to lung. The L9981-BrM only metastasized to brain. The L9981-LnM cell was only metastasized to midiastinal lymph nodes.

**Conclusions:**

A human large cell lung cancer cell model with bone, lung, brain and lymph node-specific metastasis potential was successfully established. It will be helpful to further study the molecular mechanisms and signal regulation of lung cancer organ- specific metastasis. It will be to also provide reliable cell model for developing new techniques and molecular targeting drugs of inhibiting or reversing lung cancer metastasis.

肺癌是发病率和死亡率增长最快，对人群健康和生命威胁最大的恶性肿瘤。肺癌是男性死亡率和发病率均为首位，女性发病率第二位，死亡率第一位的肿瘤^[1-7]^。近30年来，全世界肺癌发病率和死亡率不断增长，但总的治愈率却没有明显提高，5年生存率低于15%^[1, 8-14]^。肺癌高死亡率的主要原因是大约50%的患者就诊时已有远处转移，30%-40%的患者在治疗过程中发生远处转移^[15-18]^。肺癌转移是肺癌的恶性标志和生物学特征，也是导致治疗失败和死亡的最主要原因^[2-7]^。肺癌转移是一个有多基因参与调控、涉及多信号通路、多阶段、多步奏发生的复杂过程^[7, 9]^。已有的研究证明：体外培养和建立人肺癌细胞系对于研究肺癌癌变、侵袭转移、多药耐药的分子机理、生物学特征，以及开发抗肺癌新药等均有十分重要的理论和临床意义^[8, 14-18]^。研究肺癌转移的有效方法是利用具有相同遗传背景、不同转移潜能、基因和信号调节途径的肺癌细胞株作为天然对比材料^[8]^。等经过多年对肿瘤转移机制的研究，人们认识到肿瘤细胞异质性与肿瘤转移密切相关，其理论核心是：肿瘤本质上是由多克隆异质性细胞所组成，转移之所以发生，主要是由于具有高转移能力的克隆株细胞存在于肿瘤细胞群体之中。已有的研究^[19-29]^证明：肺癌与其他肿瘤一样其远处转移具有器官特异性选择。目前，国内外已经构建了前例腺癌、乳腺癌、肝癌和胰腺癌器官特异性转移细胞株^[19-29]^，但迄今国内外均无有关肺癌器官特异性转移肺癌细胞株的报道。为了筛选鉴定和建立具有器官特异性转移的人肺癌细胞株，我们应有我们实验室独有的人高转移大细胞肺癌细胞株L9981为材料，通过体内外筛选以期筛选出具有靶向器官特异性侵袭转移潜能的肺癌细胞株，为进一步的研究肺癌靶向器官特异转移提供科学可靠的细胞模型。

## 1 材料和方法

### 1.1 主要材料和仪器

① 母系高转移肺癌细胞株：L9981-Luc。L9981-Luc是应用本实验筛选的人高转移大细胞肺癌细胞株L9981，转染Luciferanse，筛选鉴定后，细胞株稳定表达Luciferanse的人高转移大细胞肺癌细胞株。②细胞培养基：RPMI-1640。③细胞培养基：购自美国Sigma公司。④精制小牛血清：购自美国Gibico公司。⑤胰蛋白酶：购自Amresco公司。⑥原代细胞培养基：购自美国JIBICO公司⑦动物活体成像仪：IVIS200型，购自美国精若真公司。⑧倒置荧光相差显微镜：尼康TE-2000型，购自日本尼康公司。

### 1.2 主要研究方法

从液氮罐取出L9981-Luc细胞株，复苏后，常规培养，等到细胞株贴壁生长良好后，将1×10^5^母系肺癌细胞株L9981-Luc接种到裸鼠皮下腹股沟皮下，每周一次应用动物活体成像仪对荷裸鼠进行活体成像，观察母系肺癌细胞株L9981-Luc在裸鼠体内的成瘤性和远处转移情况。当活体成像观察到母系肺癌细胞株L9981-Luc在远处形转移瘤后，处死裸鼠，在无菌情况下解剖出裸鼠的肺癌转移器官（肺、大脑、脊柱和纵隔淋巴结），并子无菌条件下解剖出相应的肺癌器官特异性瘤结节。在无菌条件下，在培养基中将相应的肺癌器官特异性瘤结节切成细碎片，并进行器官特异性转移的原代培养。当原代培养的器官特异性转移肺癌细胞株贴壁生长良好后，将大部分原代培养器官特异性转移肺癌细胞株在液氮中冻存，将1×10^5^原代培养器官特异性转移肺癌细胞株分别接种到裸鼠皮下腹股沟皮下，每周一次应用动物活体成像仪对荷裸鼠进行活体成像，观察原代培养器官特异性转移肺癌细胞株在裸鼠体内的成瘤性和远处转移情况。当活体成像观察到在原代培养的器官特异性转移肺癌细胞株在远处形成转移瘤后，处死裸鼠，在无菌情况下解剖出裸鼠的肺癌转移器官（肺、大脑、脊柱和纵隔淋巴结），并子无菌条件下解剖出相应的肺癌器官特异性瘤结节。在无菌条件下，在培养基中将相应的肺癌器官特异性瘤结节切成细碎片，并进行器官特异性转移的原代培养。经过如此反复的蒋器官特异性转移原代肺癌细胞株进行体内外筛选后，筛选建立成功4株人器官特异性转移肺癌细胞株（筛选流程见图 1）。

**1:**
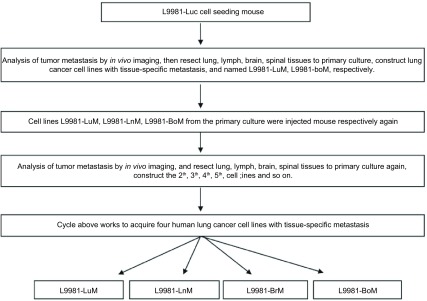
高器官靶向特异性转移潜能肺癌细胞株筛选流程图 Work flow of screening human lung cancer cell lines with high tissue-specific metastasis potential

## 2 结果

### 2.1 器官特异性转移肺癌细胞株成瘤性及器官靶向转移率比较

母系肺癌细胞株L9981-Luc为对照，4株器官特异性转移肺癌细胞株（L9981-LuM, L9981-LnM, L9981-BrM, L9981-BoM）为实验组，分别接种裸鼠，评估器官特异性转移在裸鼠体内性成瘤率和器官特异性转移百分比。每组5只裸鼠作为独立的实验，接种对照组和实验组细胞株后每周进行裸鼠活体成像，8周后解剖取得肺，淋巴，脊柱，脑组织，并解剖出器官特异性转移的转移瘤，活体成像仪分析。结果发现对照组中的5只小鼠的肺，淋巴，脊柱，脑均发生了肺癌转移，转移百分比均为100%。而实验组L9981-LuM、L9981-LnM、L9981-BrM、L9981-BoM细胞株，均具有器官特异性转移，但没有发生其他器官转移。L9981-LuM细胞株全部转移到肺，5只裸鼠均发生了肺转移瘤，转移百分比均为100%，而纵隔淋巴结，脊柱，大脑均没有发生肺癌转移。L9981-LnM细胞株全部转移到纵隔淋巴结，5只裸鼠中均发生了纵隔淋巴结转移，而肺、脊柱和大脑均为发现有肺癌转移。L9981-BrM细胞株全部转移到大脑，5只裸鼠均发生了大脑转移，而肺、淋巴结和骨均未发现有肺癌转移。L9981-BoM细胞株接种裸鼠后5只裸鼠全部发生了脊柱转移，而肺、淋巴结、和大脑均未发现肺癌转移（图 2，表 1）。

**2:**
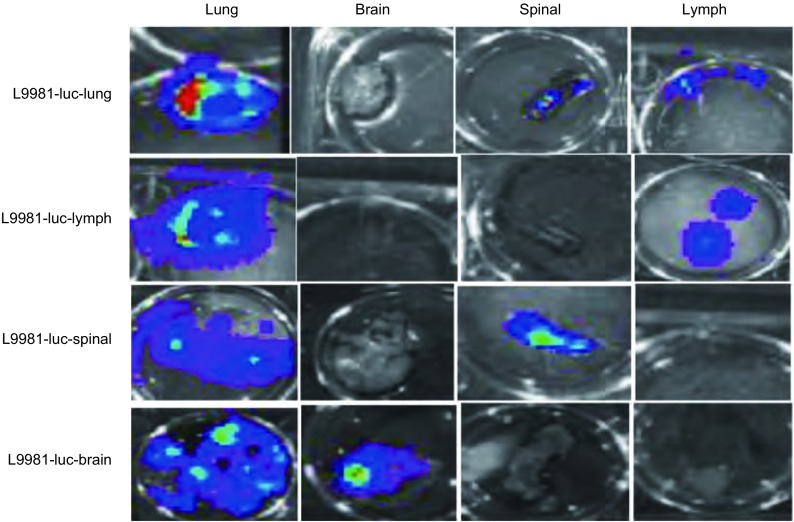
第一代原代器官特异性转移肺癌细胞株转移器官活体成像成像 The living imaging of metastasized organs planted with the first generation of organ-specific metastasis lung cancer cell line

**1:** 母系肺癌细胞株与4株器官特异性转移肺癌细胞株成瘤性及器官特异性转移率比较 comparison of tumorigenicity and organ-specific metastasis ability between the parent lung can cell line L9981 and the 4 organ-specific metastasis cell lines

Lung cancer cell lines	Planted cell number	Tumorigenicity	Organ-specific metastasis			
			Lung	Lymph	Brain	spinal
L9981-luc	5×10^6^	100%	100%	100%	100%	100%
L9981-luM	5×10^6^	100%	100%	0%	0%	0%
L9981-lnM	5×10^6^	100%	0%	100%	0%	0%
L9981-BrM	5×10^6^	100%	0%	0%	100%	0%
L9981-BoM	5×10^6^	100%	0%	0%	0%	100%

### 2.2 器官特异性转移肺癌细胞株细胞形态学

培养筛选所得人肺癌器官特异性转移潜能细胞株，分别通过100×、200×、40×显微观察其形态，与对照组细胞比较，形态均发生了不同程度的改变，其中L9981-BoM、L9981-BrM、L9981-LnM三株细胞株形态较之L9981-luc的不规则梭形均变化呈不同程度的圆形形态（图 3）。

**3:**
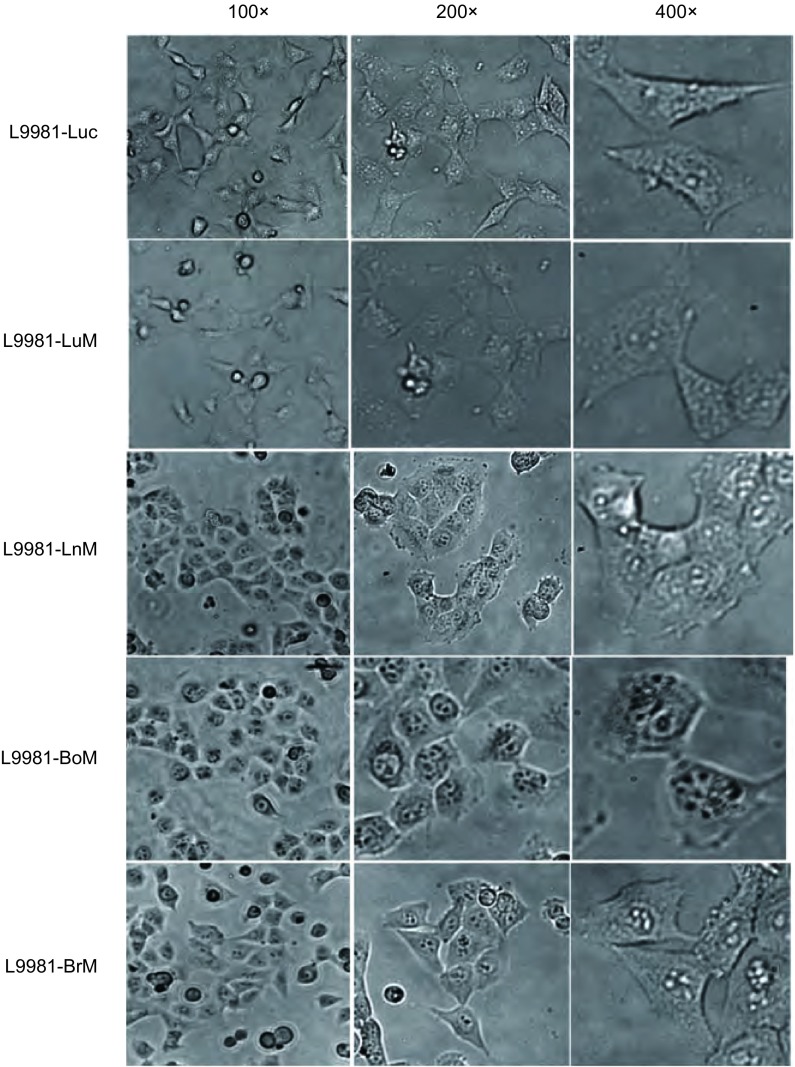
母系与器官特异性转移肺癌细胞株形态学比较 Comparison of morphology among the parent lung cancer cells and the organ-specific metastasis cells

### 2.3 人肺癌器官特异性转移细胞株转移裸鼠体内转移的活体成像结果

通过裸鼠腹股沟皮下种植筛选获得的具有靶向器官特异性转移潜能细胞，此后分别在1周、3周、6周活体成像分析其体内转移能力，发现所检测肿瘤细胞在体内普遍成瘤且发生了转移，活体成像观察的原位癌信号强弱与体内生长时间呈正相关，体内转移灶肿瘤细胞信号强弱同样与时间呈正相关（图 4）。

**4:**
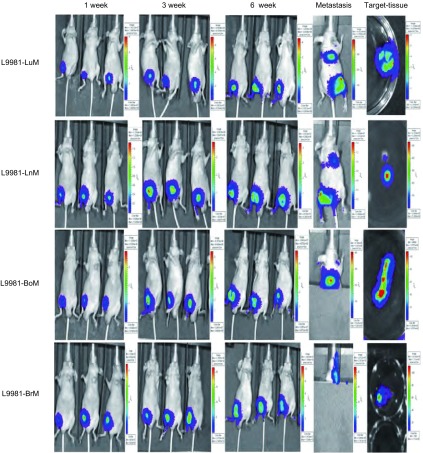
器官特异性转移肺癌细胞株裸鼠活体成像 The Living Imaging of the nude mice planted with organ-specific metastasis lung cancer cells

## 3 讨论

肺癌转移是肺癌的恶性标志和的重要生物学特征，且是造成癌症患者死亡的首要因素^[2-6]^。肿瘤的转移是由多因素参与、多基因调控，肿瘤细胞与靶器官相互作用的多阶段复杂过程^[7-10]^。每个组织、器官都有自身的结构及微环境特点，肿瘤细胞侵入靶器官就必须应对环境的压力，包括：氧气和营养的缺乏、低pH、活性氧自由基和炎症反应调节因子，经过环境的选择后，肿瘤细胞形成新的癌灶^[12-20]^。

肿瘤器官转移具有器官特异性的，不同类型肿瘤的转移对不同靶器官的亲和力往往不同，存在属于其自身的特点。前列腺癌最最常见的转移部位是骨，肝癌最常见的转移器官为肺；乳腺癌常转移到肺、骨和大脑；肺癌常转移到大脑、淋巴结、骨、肝脏和肾上腺。目前，肺癌是发病率和死亡率最高的恶性肿瘤。肺癌引发高死亡率原因主要是因为肺癌发生了其它器官的转移，比如脑、骨、脊柱、淋巴等。因此，深入研究肺癌器转特异性转移的分子机理及信号调节途径，极有可能为肺癌转移的预测、早期诊断和开发抑制/或逆转的分子靶向药物具有重要的理论意义和广阔的临床应用前景。然而，要研究肺癌器官特异性转移分子机制的最可靠方法是必须应用具有相同遗传背景、不同器官特异性转移的天然对比细胞株。正是基于上述科学问题，我们应用我们实验室的人高转移大细胞肺癌细胞株L9981为母系细胞株作研究材料，经体内外筛选，以期建立具有器官特异性转移的肺癌细胞株。

目前，国内外已经建立了多种人器官特异性转移恶性肿瘤细胞株^[22-29]^。但是，迄今为止有关肺癌器官特异性转移肺癌细胞株的筛选鉴定的研究国内外均无报道。因此，本课题组应用我们实验室建立的人高转移大细胞肺癌细胞株为材料，通过体内外的筛选鉴定，成功筛选建立了一个能特异性转移到肺、纵隔淋巴结、骨和大脑的器官特异性转移肺癌细胞模型，并将特异性转移到肺，淋巴，脑，脊柱的器官特异性转移肺癌细胞株，分别命名为L9981-LuM、L9981-LnM、L9981-BrM和L9981-BoM。随后，我们应用裸鼠肺癌自发转移动物模型，活体成像技术和病理学技术对L9981-LuM、L9981-LnM、L9981-BrM和L9981-BoM的器官特异性转移潜能进行了验证。我们的研究结果证明：与母系肺癌细胞株L9981比较，L9981-LuM、L9981-LnM、L9981-BrM和L9981-BoM肺癌细胞株除均能在裸鼠体内均成瘤外，还能特异性地转移到肺、淋巴结、大脑和骨，而L998细胞株则除了能在裸鼠体内成瘤外，还能百分之百地转移到肺、淋巴结、大脑和骨。此外，通过显微观察，4株器官特异性转移肺癌细胞株（L9981-LuM、L9981-LnM、L9981-BrM和L9981-BoM）与母系细胞株L9981比较，其形态学发生了显著改变，呈不规则圆形或椭圆型形态。我们推测，这一形态的变化可能是肺癌细胞在发生转移过程中，为了适应转移靶器官的微环境，并在靶器官形成新的癌灶，以便存活生长而发生的表型变化，但是其分子机制和细胞信号调控途径尚未完全明了，有待以后进行更深入的研究。
